# Clusters of alcohol abstainers and drinkers incorporating motives against drinking: a random survey of 18 to 30 year olds in four cities in four different continents

**DOI:** 10.3934/publichealth.2019.1.15

**Published:** 2019-01-17

**Authors:** Anne W Taylor, Bridgette M Bewick, Qing Ling, Valentina V Kirzhanova, Paulo Alterwain, Eleonora Dal Grande, Graeme Tucker, Alfred B Makanjuola

**Affiliations:** 1 Population Research and Outcome Studies, Discipline of Medicine, The University of Adelaide, South Australia, Australia; 2 School of Medicine, University of Leeds, Leeds, United Kingdom; 3 Center for Health Education, PR Ministry of Health, China; 4 Department of Epidemiology, Federal Medical Research Centre for Psychiatry and Narcology, Ministry of Health of the Russian Federation, Russia; 5 ProHumanitas Foundation, Uruguay; 6 Population Research and Outcome Studies, Discipline of Medicine, The University of Adelaide, South Australia, Australia; 7 Discipline of Medicine, The University of Adelaide, South Australia, Australia; 8 Department of Behavioural Sciences, University of Ilorin Teaching Hospital, Ilorin–Nigeria

**Keywords:** alcohol, adolescent, perceptions, motivations, survey, Moscow, Montevideo, Wuhan, Ilorin

## Abstract

**Objective:**

The aim of this analysis was to identify alcohol consumption clusters for adolescents and early adults according to attitudes to drinking, motivations against drinking and perceptions associated with alcohol.

**Method:**

Interviews were undertaken with people aged 18–34 years old living in four cities in different regions of the world. Multistage random sampling was consistent across the four cities (Ilorin (Nigeria), Wuhan (China), Montevideo (Uruguay) and Moscow (Russia)). The questionnaire was forward and back translated into relevant languages and face-to-face interviewing undertaken. The data were weighted to the population of each city. In total 6235 structured interviews were undertaken (1391 in Ilorin, 1600 in Montevideo, 1604 in Moscow and 1640 in Wuhan). Questions regarding motivation against alcohol consumption (14 items), assessing perceptions (3 items) and attitudes to drinking in certain situations (8 items) were asked of all respondents including abstainers. Factor analysis was initially undertaken to identify highly related correlated variables.

**Results:**

Cluster analysis provided a variety of clusters (Ilorin (3 clusters), Montevideo (5), Moscow (4) and Wuhan (4)). At least one cluster in each city was dominated by abstainers and another by heavy episodic drinkers. Variations by city and alcohol consumption patterns existed in regards to variables included.

**Conclusion:**

This analysis detailed the city specific motivations against drinking alcohol, and the attitudes towards alcohol consumption. Differences highlight the influence of country/city specific culture, customs, laws, societal norms and traditions.

## 1. Introduction

In understanding lifelong patterns of alcohol consumption, the early formative period of late adolescent and early adulthood, commonly acknowledged as “transition to adulthood” is seen as a crucial period of life development [1]–[4]. The behaviours and patterns associated with alcohol consumption initiated during these years are important indicators for later stages of the lifespan [2],[3]. Negative social and health outcomes associated with excess alcohol consumption in these formative years include increased road accidents, violence, injuries and ramifications associated with unplanned sex. There are also the possibility of long-term alcohol problems and dependency [3].

Many transformations, encompassing most components of life including academic, accommodation, relationships, and work/career options, occur during the transition to adulthood [3],[5]. Multi-factorial and complex beliefs, attitudes and motivations towards alcohol are also being developed. Identifying the motivating factors that influence people to drink, limit or abstain helps to understand alcohol consumption patterns during these formative years [6]–[9]. Although simplistic explanations have been highlighted, the motivations for drinking and not drinking alcohol are multifaceted. Complex interactional and situational factors have been presented in the research literature [10],[11]. Motivating factors for alcohol consumption primarily focus on aspects such as social influences, coping with stress, enhancement and conformity [6],[8],[12]. Motives for not consuming or limiting alcohol focus on harm avoidance, religion, upbringing, personal beliefs, fear of loss of control, and fear of adverse consequences [9]–[11],[13],[14]. Research highlights the important role social motives play in all facets of alcohol consumption from abstaining to heavy episodic drinking (HED), especially in the transition to adulthood age bracket of 18 to 34 years [6],[8],[12],[15]–[17]. Although negative outcomes associated with alcohol consumption are researched extensively, research into reasons and motives for abstaining or limiting alcohol consumption are less common.

Attitudes and beliefs have been shown to be important predictors of alcohol consumption especially in this age group [18] and are seen as important areas for promotion and public health campaigns. In most instances positive attitudes and beliefs towards alcohol are reflected in higher alcohol consumption. Alcohol outcome expectations are acknowledged as important predictors of alcohol consumption, especially in this age group, with more positive attitudes also related to higher alcohol consumption [12],[19],[20].

Together with motives and attitudes, socio-demographic and socio-economic variables are important indicators associated with drinking and alcohol consumption patterns [2],[21]–[26]. Age (alcohol consumption declining with age), gender (males drinking more alcohol than females), education (with differences by education level), work status (differences by employment status), and relationship status (with marriage/partnership and parenthood often reducing alcohol consumption rates), have been shown to be associated with patterns of alcohol consumption for those transitioning into adulthood.

The aim of this study was to use a methodologically-sound database of face-to-face interviews with randomly selected people aged 18 to 34 years in four cities in four different continents to determine similarities and differences in factors associated with alcohol consumption;. In addition, the study aimed to highlight variables amenable to policy, preventive and control initiates by defining distinct sub-groups of people in terms of alcohol consumption patterns and to assess and identify similarities and differences across these international cities that will assist future public health endeavors internationally and locally. Many studies of attitudes towards, and motives for and against, alcohol consumption rely on information from current drinkers only [15],[27],[28]. This study incorporates these groups but also includes non-drinker—those who have never drunk alcohol or who have quit/ceased drinking. Furthermore, many studies of adolescents and emerging adults in this regard are limited to university or college populations and are often based on USA populations [27],[29],[30]. This study uses unique, international, community, population-wide samples, representative of adolescents and young adults aged 18 to 34 years, in the four international cities. To undertake this comparison, and to determine in greater depth the alcohol consumption patterns in the four cities, cluster and factor analysis were undertaken segmenting the population into groups that are homogeneous as possible. Factor and cluster analysis in alcohol consumption provides additional in-depth reflections on pathways to effective public health prevention and control activities.

## 2. Methods

### 2.1. Study participants and sampling strategy

Four cities were chosen pragmatically based on diversity to be involved in the study. The city of Wuhan, capital of Hubei Province in China; Moscow, the capital and the largest city of Russia; Ilorin which is the administrative capital of Kwara State, Nigeria; and Montevideo, the capital of Uruguay. For each city, multistage random sampling was undertaken and was kept consistent across the four cities. In each randomly selected household, the person with the most recent birthday, aged between 18 and 34 years, and who had lived in the city for at least six months, was eligible and was invited to participate in the study.

### 2.2. Ethics

In Wuhan, ethical approval was obtained from the Hubei Provincial CDC (Hubei Provincial Society for Health Promotion and Cigarette-smoking Control, HBPHPandCCS-2014-01), in Moscow from the Ethics Committee on the NRC on Addictions, in Ilorin from the Ethics Research Committee of the University of Ilorin (UERC/ASN/2014/007) and in Montevideo from the Pro Humanities Ethics Committee.

### 2.3. Questionnaire development

The questionnaire was forward-translated into the relevant languages (e.g. English to Chinese) and back-translated (e.g. Chinese to English) to ensure the questions were conceptually and culturally equivalent between the cities. Prior to the main survey, a pilot study of 25 to 50 interviews was conducted in each city. Data collection was interviewer-administered. The average length of interviews was 15 minutes. Response rates ranged from 48.4% in Moscow to 95.0% in Ilorin. The detailed methodology and demographic profile of respondents has previously been published [31].

### 2.4. Survey questions

Each respondent was asked: 1) if they had ever consumed alcohol (excluding sips), 2) how often during the past 12 months they had drunk beer, wine, spirits (e.g., vodka, gin, whisky, brandy), and any other alcohol beverage, even in small amounts, and 3) during the past 12 months, how many alcoholic drinks they had on a typical day when they drank alcohol.

Overall quantity (i.e. usual frequency of drinking by usual number of drinks consumed per drinking occasion) was calculated by multiplying the responses to the above two questions (how much and how many) with 25 or more drinks (coded as 25), 19–24 drinks (coded as 21.5), 16–18 drinks (coded as 17), 12–15 drinks (coded as 13.5), 9–11 drinks (coded as 10), 7–8 drinks (coded as 7.5), 5–6 drinks (coded as 5.5), 3–4 drinks (coded as 3.5), 2 drinks (coded as 2), 1 drink (coded as 1) and less than 1 full drink (coded as 0.5). The annual number of drinks was calculated by multiplying the responses to the question on how many days did they drink alcohol with the response from how many drinks did they have. The variables were recoded into four drinking status groups: 0 drinks = Abstainers; > 0 but less than 365 drinks/year = Light Drinkers; 365–729 drinks/year = Moderate Drinkers; 730 or more drinks/year = Heavier drinkers.

Motivation against drinking alcohol included a question on how important the following reasons were: 1) pregnancy or trying to become pregnant (females only), 2) of taste, 3) don't like the effect it has, 4) have seen bad examples of what alcohol can do, 5) previously hurt by somebody's else drinking, 6) drinking could affect work or school performance, 7) drinking is too expensive or a waste of money, 8) religious reasons, 9) brought up not to drink, 10) had an alcohol problem or afraid of becoming an alcohol, 11) too young, 12) friends and/or family members disapprove, 13) health reasons, and 14) just not interested. Possible responses were very important, important, not very important, and not at all important. The questionnaire asked these attitude questions of current drinkers, and non-drinkers (both past drinkers and lifetime abstainers) as separate questions. The two questions were combined to make one data item for each alternative. Option 1 was asked only of females and was excluded.

General attitude to alcohol use included whether respondents agreed that 1) having a drink is one of the pleasures of life, 2) having a drink with someone is a way of being friendly, 3) there is nothing good to be said about drinking. Response categories were strongly agree, agree, neither agree nor disagree, disagree, and strongly disagree.

Respondents were also asked how many drinks people in certain situations should feel free to drink 1) as a mother, spending time with small children, 2) as a father, spending time with small children, 3) for a man out at a bar with fiends, 4) for a woman out at a bar with friends, 5) for a woman out with co-workers, 6) for a man out with co-workers, 7) for a man having dinner at home with spouse or partner, and 8) for a woman having dinner at home with spouse or partner. Responses options were: no drinks, some drinking but not enough to feel the effects (1 or 2 drinks), enough to feel the effects but not become drunk, getting drunk is sometimes alright, and getting drunk is always alright.

Demographic questions included age, sex, marital status (recoded into married/not married), highest education obtained (secondary school or less/vocational, professional, non-university/university), employment status (employed/not employed), have any children (yes/no), and currently a student (yes/no).

### 2.5. Analysis

To eliminate/reduce potential biases and to ensure that the results accurately reflected the population of interest, the data were weighted by age, sex and probability of selection. Specific demographic/census databases were used for each city. Details on specific weighting methodology are contained in a previous publication [31]. Data were analysed using Statistical Package for the Social Sciences (SPSS) version 20 for Windows (Chicago, IL).

As data on many individual motivational and attitudinal variables were collected, factor analysis was initially used to identify sets of variables for each city that were highly correlated and to simplify the analysis. Cluster analysis was then used to identify clusters and heterogeneity of groups of individuals.

Since this analysis is purely exploratory, there was no need to randomly divide the sample into two halves in order to confirm the factor structure derived from one half of the dataset on the other half. For each city, an exploratory factor analysis (EFA) was conducted. All of the above data items were entered into the analysis. Details on specific analysis for each city are detailed below. The factor scores from the analysis for each city were saved, and used as an input into a cluster analysis, along with a derived variable regarding the drinking status of the respondent. Also included in the cluster analysis were the demographic data detailed previously. Hierarchical agglomerative clustering was employed using the squared Euclidean distance measure and Ward's method to form the clusters.

## 3. Results

In total, n=6235 interviews were undertaken (1391 in Ilorin, 1600 in Montevideo, 1604 in Moscow and 1640 in Wuhan). Missing and “don't know” responses were excluded from all analyses, with the final numbers included being 1144 for Ilorin, 981 for Montevideo, 1526 for Moscow, and 1150 for Wuhan. A demographic profile of respondents for each city is presented in Supplementary The proportion having ever consumed alcohol was 33.3% (95% CI 30.9–35.9) of respondents from Ilorin, 53.4% (95% CI 51.0–55.8) for Wuhan, 86.1% (95% CI 84.3–87.7) for Moscow, and 96.4% (95% CI 95.3–97.2) for Montevideo (Table 1).

### 3.1. Factor analysis by city

#### Ilorin, Nigeria

Various extraction and rotation options were tried, with the best solution being produced by principal components extraction with an oblique rotation. When all of the items were entered into an EFA, the questions regarding general attitudes and situations formed their own factors and did not add anything to the understanding of the data. When these were removed, and the EFA considered only motivations against drinking, two factors emerged. The first factor was a motivation because of intrinsic factors (e.g. dislike for the effects of alcohol). The second factor related to motivation because of extrinsic pressures (e.g. social pressure against drinking) (Supplementary Table 2).

#### Montevideo, Uruguay

The approach to this analysis was the same as the analysis previously described for Ilorin. The final EFA considered only motivations against drinking where two factors emerged. The first factor was motivation by intrinsic personal pressures (e.g. about social pressure against drinking). The second factor was motivated by fear of effects (e.g. a dislike for the effects of alcohol) (Supplementary Table 3).

#### Moscow, Russia

When all items were entered into an EFA using maximum likelihood extraction and oblimin rotation (oblique rotation allowing for correlated factors), the questions regarding general attitudes, perceptions of situations, and motivations against drinking formed their own individual factors. Although this seems like an obvious result, it did not occur in the two previous factor analyses. The first factor was perceptions of drinking limits in different situations (e.g. for a woman out at a bar with friends), the second factor was motivation by fear (e.g. have been hurt by someone else's drinking), and the third factor was positive justification/perception of drinking (e.g. drinking is one of the pleasures of life) (Supplementary Table 4).

#### Wuhan, China

When all items were entered into an EFA, principal axis factoring produced the best extraction results, which combined with oblimin rotation produced a two factor solution where the questions regarding motivations against drinking and general attitudes/perceptions of situations formed factors. The first factor was motivation by both intrinsic and extrinsic pressures against drinking (e.g. I have had alcohol problems/are afraid of being an alcohol; my friends and/or family disapprove of me drinking). The second factor was perceptions of drinking limits in different situations (e.g. for a woman having dinner at home with her spouse or partner) and a positive justification/attitude towards drinking (e.g. having a drink with someone is a way of being friendly) (Supplementary Table 5).

### 3.2. Cluster analysis by city

#### Ilorin, Nigeria

When cluster analysis was undertaken, the dendogram and the agglomeration schedule both supported a three cluster solution encompassing 26.6%, 33.3% and 40.0% of the sample. Those clusters are detailed in Table 1. Members of Cluster 1 were more likely to be abstainers, former drinkers, older, university educated, married, employed females with children. Members of Cluster 2 were more likely to be younger than Cluster 1 and older then Cluster 3 members, unmarried with no children, who were current drinkers or HEDs. Members of Cluster 3 were all students, more likely to be young, who do not drink, are unmarried and do not have children. They were students, and they were therefore also less likely to be working. Differences by intrinsic and extrinsic pressures were apparent across the Clusters.

#### Montevideo, Uruguay

The dendogram and the agglomeration schedule both supported a five cluster solution encompassing 15.5%, 14.3%, 36.3%, 22.7% and 11.2% of the sample. These clusters are detailed in Table 2. Cluster 1 were more likely to be current alcohol drinkers or HED, younger than the other Clusters, unmarried, not employed with no children. Cluster 2 were more likely to be current drinkers, university educated, employed, females without children, and also more likely to be students. Cluster 3 were more likely to be older than the other Clusters, married, employed with children and are current drinkers or HEDs, with a lower level of education than average. They were also less likely to be students. Cluster 4 are more likely to be employed, males with no children who are HEDs. Cluster 5 were more likely to be abstainers or past drinkers with 94.7% of this cluster not drinking, and those that did were light drinkers. They were more likely to have a low level of education. Differences by intrinsic and extrinsic pressures and fears of the effects of alcohol were apparent across the Clusters.

#### Moscow, Russia

A four cluster solution was indicated by the dendogram and agglomeration schedule encompassing 23.0%, 25.2%, 36.1% and 15.6% of the sample in this case. The clusters are detailed in Table 3. Cluster 1 were more likely to be older, current drinkers or HEDs, married with children, employed, and well educated. Cluster 2 members were younger than other Clusters, unmarried, less likely to have children, less likely to be employed, or have a degree qualification. They were more likely to be lifetime abstainers although all drinker types were represented in this group. Cluster 3 were more likely to be employed, well educated, unmarried with no children who were current drinkers or HEDs. Cluster 4 are more likely to be females and less likely to be students. They were abstainers or former drinkers. Differences in perception of acceptable drinking limits, justification for drinking and motivation against drinking were apparent across the Clusters.

#### Wuhan, China

Hierarchical agglomerative clustering was employed using the squared Euclidean distance measure and Ward's method to form the clusters. This analysis did not result in clusters that discriminated drinking behaviour or attitudes. A number of different alternative extraction methods and factor solutions were tried, including reducing the factor analysis to just motivations against drinking as in Ilorin and Montevideo, but all suffered from the same problem. Eventually marital status, education status and number of children were omitted and a four cluster solution of acceptable quality was produced. The four cluster solution encompassed 36.3%, 33.2%, 21.6% and 8.8% of the sample. The clusters are detailed in Table 4. Cluster 1 were current drinkers who were employed, highly educated, and not likely to be a student. Cluster 2 were mostly non-drinkers (lifetime abstainers and former drinkers), more likely to be employed, not likely to be a student with high levels of education. Cluster 3 reflected a variety of levels of alcohol consumption patterns with the majority being current drinkers or lifetime abstainers. They were also more likely not to be married, not employed (100% students) and less likely to have children. Cluster 4 were a mixture of current drinkers and lifetime abstainers who were more likely to be not employed, non-university educated and not students. Differences in motivation against drinking and acceptable levels of drinking were apparent especially in Cluster 2.

## 4. Discussion

The results of this analysis from a random-population survey of 18 to 34 year olds in four distinct cities of the world highlighted the complexity associated with determining patterns of alcohol consumption in this important age group. Although results varied considerably across all four cities, some similarities between the cities in terms of alcohol consumption were apparent.

Two of the three clusters for Ilorin were dominated by alcohol abstainers. This reflects the relatively low overall prevalence rates of alcohol consumption in Nigeria [32]–[35], although among those who do drink alcohol, large amounts are regularly consumed [34]–[37]. The abstainer cluster, predominately students, consisted of younger people whose extrinsic motivations against drinking was dominated by religious reasons or because they were brought up not to drink alcohol. Internationally, religion and the influence of upbringing have been shown to have important considerations for abstainers and low alcohol drinkers [11],[14],[23],[30]. In Nigeria, the practice of Muslim religion is widespread [14],[32],[36] and our previous research indicated that the Muslim respondents were significantly less likely to be current drinkers [31]. In all four cities at least one of the final clusters was predominately abstainers and ex-drinkers, but religion as a motive for not consuming alcohol was only prominent in Ilorin. Internationally religious convictions have been shown to be stronger in females [12] although in our analyses the Ilorin cluster that rated religion high was not dominated by females.

**Table 1.** Results of cluster analysis for Ilorin-Nigeria.

	Cluster 1 (n=261, 26.6%)			Cluster 2 (n=327, 33.3%)			Cluster 3 (n=392, 40.0%)			Total				
	(Abstaining females)			(Heavy drinkers)			(Abstaining young students)			n=980				
Ilorin, Nigeria	n	%	AR	n	%	AR	n	%	AR	N	%	X2	Df	P value
Drinking status														
Lifetime abstainer	155	59.4	1.5	153	46.8	−3.8	235	59.9	2.3	543	55.4	24.336	6	<0.001
Former drinker	41	15.7	−0.7	70	21.4	2.6	56	14.3	−1.9	167	17.0			
Current drinker	53	20.3	−1.4	82	25.1	0.9	94	24.0	0.4	229	23.4			
Heavy episodic drinker	12	4.6	0.4	22	6.7	2.8	7	1.8	−3.1	41	4.2			
Age (mean years)	30.05			24.74			22.29							
Gender														
Male	68	26.1	−8.3	195	59.6	5.1	209	53.3	2.6	472	48.2	72.503	2	<0.001
Marital Status														
Married	259	99.2	30.3	10	3.1	−12.1	1	0.3	−15.6	270	27.5	917.453	2	<0.001
Employment Status														
Employed	199	76.0	12.7	166	50.8	3.6	55	14.0	−14.9	420	42.8	258.616	2	<0.001
Highest level of education														
Secondary school or less	152	58.2	−0.1	215	65.7	3.2	207	52.8	−3	574	58.6	162.965	4	<0.001
Vocational/professional/Non–university tertiary education	37	14.2	−5.4	50	15.3	−5.8	177	45.2	10.5	264	26.9			
University degree or higher	72	27.6	7	62	19.0	2.8	8	2.0	−9	142	14.5			
Children?														
Yes	248	95.0	25.9	40	12.2	−9.1	20	5.1	−14.5	308	31.4	672.53	2	<0.001
Student?														
Yes	3	1.1	−15.3	6	1.8	−17.6	392	100.0	30.7	401	40.9	943.37	2	<0.001
Factor 1 – Intrinsic pressures (mean) ^a^	0.34↓			0.06			0.02							
Factor 2 – Extrinsic pressures (mean)	0.26			0.34			−0.09↑							

Note: The weighting of the data can lead to rounding discrepancies and totals not adding. (a) Uses Harmonic Mean Sample Size= 318.013; the group sizes are unequal. The harmonic mean of the group sizes is used; Type I error levels are not guaranteed. ↓ Less important ↑ More important.

**Table 2.** Results of cluster analysis for Montevideo-Uruguay.

	Cluster 1			Cluster 2			Cluster 3			Cluster 4			Cluster 5							
	(n=237, 15.5%)			(n=217, 14.3%)			(n= 554, 36.3%)			(n=347, 22.7%)			(n=170, 11.2%)			Total				
	(Young drinkers)			(Student drinkers)			(Older married HED)			(Male HED)			(Abstainers)			(n = 1525)				
	n	%	AR	n	%	AR	n	%	AR	n	%	AR	n	%	AR	n	%	X2	Df	P value
Drinking status																				
Lifetime abstainer	0	0.0	−2.8	0	0.0	−2.7	2	0.4	−4.3	0	0.0	−3.6	40	23.5	17.6	42	2.8	1400.6	16	<0.001#
Former drinker	0	0.0	−5.1	4	1.8	−3.8	3	0.5	−8.4	1	0.3	−6.2	121	71.2	31.2	129	8.5			
Very light drinker	7	3.0	0	5	2.3	−0.6	17	3.1	0.2	7	2.0	−1.2	9	5.3	1.9	45	3.0			
Current drinker	165	69.6	1.2	187	86.2	6.7	422	76.2	6.3	234	67.4	0.6	0	0.0	−19	1008	66.1			
Heavy episodic drinker	65	27.4	3.2	21	9.7	−4	110	19.9	0.1	105	30.3	5.6	0	0.0	−6.9	301	19.7			
Age (mean years)	21.64			25.60			28.12			25.41			25.55							
Gender																				
Male	110	46.2	−1.1	3	1.4	−15.3	243	43.9	−3.3	330	95.1	19.3	69	40.6	−2.5	755	49.5	503.12	4	<0.001
Marital Status																				
Married	7	3.0	−15.0	104	47.7	0	420	75.8	16.6	103	29.7	−7.6	93	54.7	2.0	727	47.6	414.26	4	
Employment																				
Employed	0	0.0	−24.7	196	90.3	7.5	404	72.9	2.8	341	98.6	13.7	102	60.0	−2.5	1043	68.4	718.08	4	
Highest level of education																				
Secondary school or less	122	51.5	−2.1	56	25.7	−10.3	398	71.8	8.5	184	53.2	−1.9	118	69.0	3.2	878	57.5	243.48	8	<0.001
Vocational/prof/Non–uni	102	43.0	3.4	94	43.1	3.2	130	23.5	−6.3	138	39.9	2.8	48	28.1	−1.6	512	33.6			
University degree	13	5.5	−2	68	31.2	12.5	26	4.7	−4.4	24	6.9	−1.5	5	2.9	−2.9	136	8.9			
Children?																				
Yes	21	8.9	−11.5	36	16.5	−8.5	487	87.9	26.8	0	0.0	−18.4	111	65.3	6.3	655	42.9	927.55	4	<0.001
Student?																				
Yes	158	66.7	11.1	171	78.4	14.5	27	4.9	−18.7	136	39.2	1.8	43	25.3	−2.8	535	35.1	515.63	4	<0.001
Factor 1–Intrinsic pressure (mean) ^a^	−0.03			0.13			0.18			−0.02			−0.37↑							
Factor 2–Fear of effects (mean)	0.11			−0.24↑			0.07			0.14			−0.29↑							

Note: The weighting of the data can lead to rounding discrepancies and totals not adding. (a) Uses Harmonic Mean Sample Size =257.986; the group sizes are unequal. The harmonic mean of the group sizes is used; Type I error levels are not guaranteed. # 1 cell < 5. ↓ Less important ↑ More important.

**Table 3.** Results of cluster analysis for Moscow-Russia.

	Cluster 1			Cluster 2			Cluster 3			Cluster 4			Total (n= 1066)					
	(n=245, 23.0%)			(n=269, 25.2%)			(n=385, 36.1%)			(n=167, 15.6%)								
	(Older drinkers)			(Students)			(Current drinkers)			(Abstainers, females)								
	n	%	AR	n	%	AR	n	%	AR	N	%	AR	n	%	X2	Df	P value	
Drinking status																		
Lifetime abstainer	0	0.0	−6.2	64	23.8	7.9	0	0.0	−8.6	52	31.3	9.2	116	10.9	528.728	12	<0.001	Cells < 5.
Former drinker	1	0.4	−5.4	21	7.8	−0.9	5	1.3	−6.7	71	42.8	16.3	98	9.2				
Very light drinker	0	0.0	−0.5	1	0.4	1.7	0	0.0	−0.8	0	0.0	−0.4	1	0.1				
Current drinker	2128	86.5	6.0	173	64.3	−2.9	334	86.8	8.4	40	24.1	−14.6	759	71.3				
Heavy episodic drinker	32	13.1	2.9	10	3.7	−3.3	46	11.9	3.0	3	1.8	−3.4	91	8.5				
Age (mean years)	30.94			20.61			26.54			28.65								
Gender																		
Male	132	53.9	1.3	131	48.7	0.3	205	53.2	3.3	43	25.7	−6.2	511	47.9	40.820	3	<0.001	
Marital Status																		
Married	207	84.5	16.4	11	4.1	−13.7	85	22.1	−8.7	118	70.7	9.0	421	39.5	465.430	3	<0.001	
Employment																		
Employed	244	99.6	11.0	57	21.2	−21.4	370	96.1	13.2	95	57.2	−4.6	766	71.9	564.995	3	<0.001	
Highest level of education																		
Secondary school or less	11	4.5	−5.4	103	38.4	12.1	27	7.0	−5.7	23	13.9	−0.6	164	15.4	269.003	6	<0.001	
Vocational/prof/Non–uni	105	42.9	−0.3	155	57.8	5.4	143	37.1	−1.8	62	37.3	−1.8	435	40.9				
University degree or higher	129	52.7	4.3	10	3.7	−14.3	215	55.8	7.5	81	48.8	2.3	435	40.9				
Children?																		
Yes	245	99.6	25.3	7	2.6	−12.3	3	0.8	−16.8	97	58.4	7.6	352	33.0	834.912	3	<0.001	
Student?																		
Yes	1	0.4	−10.5	269	100.0	31.7	0	0.0	−14.7	11	6.6	−6.3	281	26.4	1007.935	3	<0.001	
Factor 1 – Situations (mean) ^a^	0.28↑			−0.07			0.33↑			−0.58↓								
Factor 2 – Motivation by fear (mean)	0.17↓			−0.19↑			0.20↓			−0.31↑								
Factor 3 – Positive justification (mean)	−0.37↑			0.18			−0.45			0.63↓								

Note: The weighting of the data can lead to rounding discrepancies and totals not adding; (a) Uses Harmonic Mean Sample Size = 243.870; the group sizes are unequal. The harmonic mean of the group sizes is used; Type I error levels are not guaranteed. ↓ Less important ↑ More important.

**Table 4.** Cluster analysis for Wuhan-China.

	Cluster 1			Cluster 2			Cluster 3			Cluster 4								
	(n=412, 36.3%)			(n=376, 33.2%)			(n=245, 21.6%)			(n=100, 8.8%)			Total					
	(Drinkers)			(Abstainers)			(Abstainers/former)			(Unemployed)			(n = 1133)					
	n	%	AR	n	%	AR	n	%	AR	n	%	AR	n	%	X2	Df	P value	
Drinking status																		
Lifetime abstainer	36	8.8	−17.6	324	85.9	20.6	84	34.1	−3.2	45	44.6	0.3	489	43.1	667.791	12	<0.001	
Former drinker	5	1.2	−5.9	48	12.7	5.1	25	10.2	2.0	4	4.0	−1.3	82	7.2				
Very light drinker	10	2.4	−0.7	4	1.1	−2.6	18	7.3	4.7	1	1.0	−1.2	33	2.9				
Current drinker	321	78.1	18.2	1	0.3	−20.3	113	45.9	1.2	48	47.5	1.1	483	42.6				
Heavy episodic drinker	39	9.5	6.6	0	0.0	−5.0	6	2.4	−1.6	3	3.0	−0.7	48	4.2				
Age (mean years)	26.13			26.30			20.41			26.67								
Gender																		
Male	247	60.0	4.5	185	49.2	−0.9	121	49.4	−0.6	27	27.0	−5.1	580	51.2	36.990	3	<0.001	
Marital Status																		
Married	199	48.7	2.7	212	56.4	6.3	8	3.3	−14.3	70	70.0	5.6	489	43.3	220.003	3	0.008	
Employment																		
Employed	412	100.0	16.3	376	100.0	15.2	15	6.1	−25.2	0	0	−16.3	803	70.9	1064.784	3	0.014	
Highest level of education																		
Secondary school or less	37	9.0	1.2	34	9.0	1.1	0	0.0	−5.1	17	16.8	3.6	88	7.8	76.331	6	0.015	
Vocational/professional/Non–university	238	58.2	3.0	196	52.1	−0.0	99	40.4	−4.2	59	58.4	1.3	592	52.3				
University degree or higher	134	32.8	−3.7	146	38.8	−0.5	146	59.6	7.1	25	25.8	−3.3	451	39.9				
Children?																		
Yes	147	36.2	0.8	163	44.1	4.6	11	4.7	−10.9	65	65.0	6.7	386	34.8	147.998	3	0.006	
Student?																		
Yes	0	0.0	−13.4	0	0.0	−12.5	245	100.0	33.7	0	0.0	−5.5	245	21.6	1133.000	3	0.005	
Factor 1 – Intrinsic/Extrinsic (mean)(a)	0.25			−0.29↑			0.14			0.05								
Factor 2 –Situations (mean)	−0.45↑			0.36↓			0.12			0.13								

Note: The weighting of the data can lead to rounding discrepancies and totals not adding. (a) Uses Harmonic Mean Sample Size= 208.716; the group sizes are unequal. The harmonic mean of the group sizes is used; Type I error levels are not guaranteed. ↓ Less important ↑ More important.

The other abstainers and former drinker cluster found in the Ilorin sample was dominated by married females. Less important for this cluster was motivation by intrinsic and extrinsic pressures such as disliking the effect of alcohol. This again reflects the dominant culture in Nigeria where females not consuming alcohol is the societal norm [32],[35],[37],[38], although this presumption is being challenged in recent times [38]. The third cluster for Ilorin was dominated by married men who were heavy drinkers again reflecting the dominant alcohol culture found in Nigeria [32],[35],[37]–[39].

In the Montevideo analyses, five clusters were apparent. As a reflection of the higher alcohol consumption prevalence rates found in Montevideo [17],[25], only one cluster featured abstainers or former drinkers and only represented 11.2% of the total Montevideo sample. Although the mean age of this cluster (26 years) was not the lowest, this cluster's main motivation against drinking was because they were too young, because friends and family disapprove of their drinking and because they were brought up not to drink. The minimum legal age to purchase alcohol in Uruguay is 18 years, although there is no law related to age of consumption of alcohol, so the young age reflection must be self-perceived. Also included in this cluster, and which may explain the inclusion of the first factor, was the motivation against alcohol because of fear which included having seen bad examples of what alcohol can do, and having been hurt by somebody else's drinking. It should be noted that the majority in this cluster were ex-drinkers rather than abstainers per se, perhaps indicating previous bad experiences. Other research has shown the prominence of ‘being brought up not to drink’ as an important factor for abstainers [14]. Of note, the family/friends influence was more highly rated in the Montevideo factors than in other cities. Family and/or friend disapproval, in particular, is a common reason for limiting alcohol consumption, especially among younger people [11],[13],[17].

The cluster patterns for Moscow consisted of one cluster where abstainers and former drinkers represented over 70% of the cluster group. Again, based on the high overall alcohol consumption rates [25],[40],[41], this cluster was smaller in number of respondents and was dominated by females. Higher rates of alcohol consumption for males is frequently acknowledged in Russia [39],[42]. Also important in this cluster was the motivation against alcohol consumption because of fear such as they had been hurt by somebody else's drinking, because of health reasons and because they had seen bad examples. This high rating of health reasons (whether current, perceived or possibly into the future) is unusual in this age group as the limiting of alcohol consumption because of health reasons are usually associated with older persons [13]. This could be the result of intensive alcohol reduction programs implemented in recent years by public health agencies that has resulted in changes in alcohol use [43],[44]. It has been suggested that alcohol campaigns aimed at limiting consumption should concentrate on broader issues than health outcomes, instead incorporating social and community issues such as crime rates, driving accidents, and economics [6]. Not surprising for Moscow was the domination of HEDs in each of the remaining two clusters. In both of these clusters the positive justification represented by a belief that having a drink is one of the pleasures of life was more common in line with previous studies [19],[45]. This factor was less likely to be present in the abstainer cluster which again has previously been reported [19],[45].

It has been argued that alcohol consumption patterns in China do not always follow patterns of Western countries [28],[46]. Our results indicate this to be true with both initially the factor analysis, and then the cluster analysis, producing different patterns and results in Wuhan compared to the other three cities. While China has a long history of alcohol consumption [46], overall prevalence rates have been relatively low until recent times, especially for females. China's frequency of drinking alcohol and amount consumed at a population level is now approaching other countries levels [25],[46]–[48]. The transition is more notable in the age group of focus in this study [46]. Unsurprisingly, based on the lower overall alcohol prevalence rate, three of the four final clusters had higher rates of abstainers and former drinkers. The clusters consisted of two distinct groups with opposite levels of importance for the factors included. All the clusters had high levels of education. For the largest abstaining group (Cluster 2), the intrinsic/extrinsic factor associated with the motivation against alcohol ‘because they had alcohol problems’ or ‘were afraid they would become an alcoholic’, was stronger for this cluster.

Although it would be expected that gender would play an important part in these results, separate clusters dominated by males and females were only found in two of the Montevideo clusters, one cluster for Wuhan and none for the other cities. In this instance the Montevideo male dominant cluster tended to be unmarried, employed males who were heavy drinkers and in the separate cluster females were more likely to be educated, employed and current drinkers. In the final Wuhan cluster, females represented 73% of the sample. Other important descriptive variables included, not being employed and not being students, indicating perhaps a stay-at-home mother (with over 65% having children). While different patterns of alcohol consumption have been historically important, as female's alcohol consumption rates increasingly head towards convergence with males, especially in this age group, it is expected that different patterns would become less prominent [24], although this merging of the female and male alcohol overall consumption rate is contested in the literature [21]. Of note, Loose and Acier [6] report lack of differences in motives for each sex in their French study of students as did Mezquita et al. [16] in their study of Spanish students.

Weaknesses associated with this study include the fact that these analyses are limited to cross sectional studies with no cause or effect or long-term trends implied. We also did not take into account the economic and development status of each city/country, nor investigate relationships with other risk factors (such as smokers and drug intake) which are factors that are an important consideration for the age group we studied [2]. In addition, limiting our alcohol consumption measures to one measure (frequency/quantity) rather than multiple dimensions could be seen as a weakness. Furthermore, the lack of details on personality traits, an important consideration when assessing alcohol consumption patterns, is acknowledged [16],[29]. It is also acknowledged that alcohol consumption patterns within a community are a reflection of that location's political structure, laws and regulations, societal norms and traditions, dominance of anti-alcohol religious convictions, average income levels and economic standings, as well as motives, behaviors and beliefs. Many of these aspects were not addressed in this study. Additionally, it is generally accepted, in theory, that attitudes determine behaviours. Theory also has it that in some circumstances behaviours determine attitudes, (e.g. this is partly what smoking bans hope to achieve). Either way, the theory of factorial causation dictates that attitudes and behaviours should not be analysed in the same exploratory factor analysis (EFA), which we did undertake.

This study, in its rigorous attention to pre-survey detail and cultural differences, has endeavored to overcome any major shortcomings. The strengths of this study include the diversity of the cities studied, the focus on the limited age range, the relatively large sample size, high response rates and the use of probability-based sampling methodology (stratified, clustered, systematic) in each city. In addition, often alcohol consumption is not recorded in many official statistics and this self-report methodology has been able to incorporate all levels of consumption. An additional strength of the study is the inclusion of both abstainers and ex-drinkers, a group often omitted from these types of analyses. Weighting of the data allowed for the estimates to be representative of the general population. A further strength was the use, as far as possible, of comparable measures of alcohol consumption and demographic variables and similar methodological aspects such as sample selection, protocols, and administration. This included city specific interviewing. The involvement of local communities and language specific interviewing with translation and back translation of all questionnaires are also strengths of the study. The use of pre-designed epidemiological-sound methodology, rather than post-data collection manipulation of already collected data, is seen as an additional strength.

This research, addressing the need for more geographical based, methodologically-comparable studies especially in low and middle income countries [3],[48], has gone someway in satisfying Bloomfield et al's [49] call for descriptive epidemiological alcohol-related research across multiple regions of the world and calls for more studies on motivation associated with alcohol consumption and abstaining [10],[13]. Future research could address the effects of specific alcohol related policies, guidelines, legislation and economic development in each of these cities. In addition, the role and pattern of social interactions has been highlighted as an important component associated with alcohol consumption in this age group and further research into this aspect of life would be beneficial.

In conclusion, specific groupings and characteristics regarding attitudes to, and motives against, alcohol consumption are likely to exist. This study identified up to five groups for each city examined, which provides greater understanding of the patterns of alcohol consumption in these communities and increased understanding of these key motivations for drinking alcohol. Knowledge and awareness of these patterns highlights opportunities to provide more targeted preventative education and communicative strategies. This research highlights how motivations and behaviours associated with alcohol consumption are closely connected to specific countries and cultures and the importance of socio-demographic indicators.
