# Supplementary material for: Clusters of alcohol abstainers and drinkers incorporating motives against drinking: a random survey of 18 to 30 year olds in four cities in four different continents
Source: AIMS Public Health. 2019 Jan 17;6(1):15–33. doi: 10.3934/publichealth.2019.1.15 (PMC6433617; doi:10.3934/publichealth.2019.1.15)
Supplement: Supplementary file 1 [file publichealth-06-01-015-s001.pdf]

AIMS Public Health

AIMS Public Health, 6(1):15–33.
DOI: 10.3934/publichealth.2019.1.15
Received: 12 September 2018
Accepted: 17 December 2018
Published: 17 January 2019

http://www.aimspress.com/journal/aimsph

Research article

Clusters of alcohol abstainers and drinkers incorporating motives against drinking: a random survey of 18 to 34 year olds in four cities in four different continents

Anne W T aylor[1,*], Bridgette M Bewick[2], Qing Ling[3], Valentina V Kirzhanova[4], Paulo Alterwain[5], Eleonora Dal Grande[6], Graeme Tucker[7] and Alfred B Makanjuola[8]

[1] Population Research and Outcome Studies, Discipline of Medicine, The University of Adelaide, South Australia, Australia
[2] School of Medicine, University of Leeds, Leeds, United Kingdom
[3] Center for Health Education, PR Ministry of Health, China
[4] Department of Epidemiology, Federal Medical Research Centre for Psychiatry and Narcology, Ministry of Health of the Russian Federation, Russia
[5] ProHumanitas Foundation, Uruguay
[6] Population Research and Outcome Studies, Discipline of Medicine, The University of Adelaide, South Australia, Australia
[7] Discipline of Medicine, The University of Adelaide, South Australia, Australia
[8] Department of Behavioural Sciences, University of Ilorin Teaching Hospital, Ilorin−Nigeria

* **Correspondence**: Email: Anne.Taylor@adelaide.edu.au; Tel: +610883131211.

Supplementary Table 1. Demographic characteristics of respondents.

	Ilorin	Montevideo	Moscow	Wuhan
Total (n)	1391	1600	1604	1657
Sex (% male)	48.2	49.5	48.0	51.2
Age (mean years)	25.1	25.8	26.2	25.2
Education (%)				
School	58.6	57.5	15.4	7.6
University	14.5	8.9	40.8	40.0
Marital status (%)	27.5	47.6	26.3	21.8
Student (%)	40.9	35.1	26.3	21.8
Employed (%)	42.8	68.4	72.0	70.8
Children (%)	31.4	42.9	33.1	34.7

Supplementary Table 2. Factor analysis pattern matrix—Ilorin, Nigeria[a].

	Component	
	1	2
	Intrinsic	Extrinsic
Motivation against – Because:	0.796	0.001
− I don't like the effect it has on me		
− Drinking could affect my work or school performance	0.674	0.217
− I have had alcohol problems/are afraid of becoming an alcoholic	0.665	−0.123
− I have seen bad examples of what alcohol can do	0.632	0.224
− I am too young	0.601	0.131
− Of the taste	0.589	−0.191
− I have been hurt by someone else's drinking	0.514	−0.166
− Drinking is too expensive or a waste of money	0.507	0.042
− For health reasons	0.459	0.175
Motivation against – Because:	0.152	0.800
− Of religious reasons		
− I was brought up not to drink	0.165	0.798
− My friends and/or family disapprove of me drinking	0.388	0.510
− I am just not interested	−0.215	0.471

Note: Extraction Method: Principal Component Analysis. Rotation Method: Oblimin with Kaiser Normalization. a. Rotation converged in 7 iterations.

Supplementary Table 3. Factor analysis pattern matrix—Montevideo, Uruguay[a].

	Component	
	1	2
	Intrinsic	Fear of effects
Motivation against − Because:	0.807	−0.177
− I am too young		
− My friends and/or family disapprove of me drinking	0.746	−0.112
− I was brought up not to drink	0.634	−0.014
− For health reasons	0.615	0.030
− I have had alcohol problems/are afraid of becoming an alcoholic	0.576	0.099
− Of religious reasons	0.513	0.044
− Drinking is too expensive or a waste of money	0.435	0.257
− I am just not interested	0.295	0.275
Motivation against – Because:	0.054	0.760
− I have seen bad examples of what alcohol can do		
− I have been hurt by someone else's drinking	0.036	0.714
− I don't like the effect it has on me	0.027	0.674
− Drinking could affect my work or school performance	0.255	0.495
− Of the taste	−0.143	0.467

Note: Extraction Method: Principal Component Analysis; Rotation Method: Oblimin with Kaiser Normalization. A.Rotation converged in 6 iterations.

Supplementary Table 4. Factor analysis pattern matrix—Moscow, Russia[a].

	Factor		
	1	2	3
	Situation	Fear	Justification
Please tell me how much a person in that situation should drink?	0.878	−0.091	0.083
For a woman out at bar with friends			
For a woman out with co−workers	0.867	−0.049	0.100
For a woman having dinner at home with her spouse or partner	0.767	0.016	0.032
For a man at out at a bar with friends	0.762	−0.042	−0.077
For a man having dinner at home with his spouse or partner	0.716	0.063	−0.003
For a man out with co−workers	0.713	0.019	−0.064
As a father, spending time with small children	0.348	0.098	−0.083
As a mother, spending time with small children	0.296	0.010	−0.028

Continued on next page

	Factor		
	1	2	3
	Situation	Fear	Justification
Motivation against – Because:	0.029	0.684	0.090
− I have been hurt by someone else's drinking			
− For health reasons	−0.146	0.618	0.064
− I have seen bad examples of what alcohol can do	0.108	0.600	−0.071
− I don't like the effect it has on me	0.102	0.595	−0.102
− I have had alcohol problems/are afraid of becoming an alcoholic	−0.113	0.591	0.209
− Drinking could affect my work or school performance	−0.001	0.578	0.021
− My friends and/or family disapprove of me drinking	−0.042	0.561	−0.067
− Of the taste	0.036	0.473	−0.133
− I am too young	−0.027	0.470	−0.186
− I am just not interested	0.091	0.411	−0.357
− Drinking is too expensive or a waste of money	0.092	0.411	−0.252
− Of religious reasons	0.131	0.396	0.024
− I was brought up not to drink	0.180	0.390	−0.187
Please tell me whether you are agreeable to statement:	0.006	−0.008	0.853
− Drinking is one of the pleasures of life			
− Having a dink with someone is a way of being friendly	−0.012	0.014	0.792
−There is nothing good to be said about drinking	0.074	0.016	−0.644

Note: Extraction Method: Maximum Likelihood. Rotation Method: Oblimin with Kaiser Normalization. a. Rotation converged in 5 iterations.

Supplementary Table 5. Factor analysis pattern matrix—Wuhan, China[a].

	1	2
	Intrinsic/Extrinsic	Situation
Motivation against – Because:		
− I have had alcohol problems/are afraid of becoming an alcoholic	0.753	0.026
− My friends and/or family disapprove of me drinking	0.704	0.054
− I am too young	0.700	0.022
− I was brought up not to drink	0.677	−0.050
− Of religious reasons	0.676	0.051
− I have been hurt by someone else's drinking	0.659	0.081
− Drinking is too expensive or a waste of money	0.655	0.017
− Drinking could affect my work or school performance	0.637	0.022
− I have seen bad examples of what alcohol can do	0.606	−0.015
− For health reasons	0.554	0.029
− I don't like the effect it has on me	0.550	−0.042
− Of the taste	0.487	0.063
− I am just not interested	0.389	−0.143

Continued on next page

	1 Intrinsic/Extrinsic	2 Situation
Please tell me how much a person in that situation should drink?		
For a woman having dinner at home with her spouse or partner	−0.104	−0.802
For a man having dinner at home with his spouse or partner	−0.105	−0.792
For a woman out at bar with friends	−0.020	−0.759
For a woman out with co−workers	−0.062	−0.740
For a man out with co−workers	−0.001	−0.719
For a man at out at a bar with friends	0.016	−0.709
As a mother, spending time with small children	−0.010	−0.527
As a father, spending time with small children	−0.029	−0.515
Please tell me whether you are agreeable to statement:		
− Having a drink with someone is a way of being friendly	−0.181	0.194
− Drinking is one of the pleasures of life	−0.158	0.189
− There is nothing good to be said about drinking	0.100	−0.144

AIMS Press

© 2019 the Author(s), licensee AIMS Press. This is an open access article distributed under the terms of the Creative Commons Attribution License (http://creativecommons.org/licenses/by/4.0)
